# Attitudes and perceptions regarding antimicrobial use and resistance among medical students in Central China

**DOI:** 10.1186/s40064-016-3454-0

**Published:** 2016-10-12

**Authors:** Kun Yang, Dongfang Wu, Fei Tan, Shaojun Shi, Xianxi Guo, Qing Min, Xiaolian Zhang, Hong Cheng

**Affiliations:** 1 Department of Pharmacy, Zhongnan Hospital of Wuhan University, Wuhan, 430071 China; 2 The Second Clinical College, Tongji Medical College of Huazhong University of Science and Technology, Wuhan, China; 3 Department of Pharmacy, Union Hospital, Tongji Medical College of Huazhong University of Science and Technology, Wuhan, China; 4 Department of Pharmacy, Renmin Hospital, Wuhan University, Wuhan, China; 5 Department of Pharmacy, Hubei University of Science and Technology, Wuhan, China; 6 Department of Immunology, Medical College of Wuhan University, Wuhan, China

**Keywords:** Antimicrobial, Infection, Microbial drug resistance, Medical students, Survey

## Abstract

**Objectives:**

Senior medical students, who are future doctors, should be prepared to use antimicrobials appropriately and will be important partners in antimicrobial stewardship. This survey was designed to investigate the attitudes and perceptions of senior medical students regarding antimicrobial use and resistance.

**Methodology:**

We performed a multi-center survey involving a questionnaire handed out to all fourth year medical students from five representative teaching hospitals in Central China. The survey was completed within 1 month (October to November, 2015). Antimicrobial stewardship programs were taught in all of the teaching hospitals, yet only part of the respondents took part in it.

**Results:**

A total of 611 out of 728 students completed our survey. The majority of the respondents (92 %) believed that inappropriate use of antimicrobials causes antimicrobial resistance and agreed with the importance of a strong knowledge of antimicrobials in their medical careers. Most students (67 %) rated their education concerning antimicrobial use and resistance as useful or very useful, but only 25 % recalled having courses on this subject. The overall mean number of correct answers on a section of 11 knowledge questions was 3.78 (standard deviation 1.57, P value for score between hospitals <0.001).

**Conclusions:**

We should make an effort to optimize curriculum system in Chinese institutions, and this may contribute to making our future doctors better prepared for antimicrobial stewardship and prudent antimicrobial prescribing.

**Electronic supplementary material:**

The online version of this article (doi:10.1186/s40064-016-3454-0) contains supplementary material, which is available to authorized users.

## Background

Antimicrobial resistance poses a catastrophic threat to the entire world today. Antibiotic-resistant microorganisms are recognized as “nightmare bacteria” by world health leaders (Review on Antimicrobial Resistance 2016). Although widespread use of antimicrobials brought millions of people back to health, it also led to the emergence and spread of antimicrobial resistance across the world (Wilcox et al. 2009). This evolving public health issue is driven by both the irrational use of antimicrobials for human health and the inadequate available measures to control the spread of infections (World Health Day 2011). While antimicrobial medicines are the mainstay of treatment for bacterial infections, new products coming to the market have not kept pace with the increasing need for improvements in antimicrobial treatment (World Health Organization 2012). Antimicrobial stewardship programme (2014) is a series of policies introduced by the government for improving antimicrobial prescribing behaviors of doctors. It mainly includes leadership commitment and accountability, and key supporting groups implement policies for optimal antimicrobial use and interventions to avoid antimicrobial resistance. The policies support the implementation of facility-specific treatment recommendations. The antimicrobial stewardship programs were recommended by the Centers for Disease Control and Prevention (CDC) for improving the antimicrobial prescription in hospitals (Centers for Disease Control and Prevention 2013; Centers for Disease Control and Prevention 2014). The World Health Organization (WHO) has recommended training for medical undergraduates regarding the prudent prescription of antimicrobials (World Health Organization 2012). It is necessary that our future doctors are better equipped with better knowledge of antimicrobial use and resistance.

Unlike the senior doctors or infectious specialists who have a large amount of experience in anti-infection treatment, the junior doctors usually have limited knowledge and skills to reduce the potential risk of antimicrobial resistance (Charani et al. 2010). Therefore, antimicrobial stewardship efforts should be made to standardize the prescribing behaviors of our future doctors (Hecker et al. 2003; Owens et al. 2004; Paterson 2006). However, thus far, less attention has been given to future doctors during their medical college in China. Studies of medical students from other countries have documented students’ perceptions on antimicrobial stewardship and students’ feelings about their education regarding antimicrobial use and resistance (Abbo et al. 2013; Afzal Khan et al. 2013; Dyar et al. 2013; Ibia et al. 2005; Luther et al. 2013; Minen et al. 2010; Pulcini et al. 2015; Thriemer et al. 2013). Previous studies on antimicrobial education in the United States reported that there is an obvious gap among the medical students from different US medical schools in terms of choosing study references, preparedness for prescribing antimicrobials, and perceptions of knowledge regarding antimicrobials (Abbo et al. 2013; Ibia et al. 2005; Minen et al. 2010). However, very few studies investigated that of Chinese medical students (Huang et al. 2013; Li et al. 2012; Lv et al. 2014). The attitudes and perceptions of the Chinese senior medical students concerning these issues also warrant investigation.

This study aimed to determine the Chinese fourth year medical students’ attitudes and perceptions regarding antimicrobial use and resistance. In China, medical students will be authorized to prescribe antimicrobials after training and examination. The medical undergraduates should generally finish their 5-year training with 3 years of academic curriculum cand 2 years of clinical practice before graduating. The medical education offers the students specialized curricula including pharmacology and microbiology, but not courses of antimicrobial use and resistance. Management and prescribing of antimicrobials are generally taught by senior physicians during the latter 2 years of college. The results of our study may be useful to enable the implementation of more courses and training in antimicrobial stewardship for medical college students, thereby improving the future doctors’ performances on antimicrobial prescription in hospitals.

## Methods

### Study design and population

In China, junior doctors will be permitted to prescribe antimicrobials when they finish antimicrobial stewardship training and pass the final examination. The training focus on knowledge of infectious diseases and antimicrobial prescribing. In our study, a cross-sectional, multi-center, anonymous questionnaire was designed to evaluate fourth-year medical students’ attitudes and perceptions regarding antimicrobial use and resistance in Central China, including how they feel about their education with respect to appropriate use of antimicrobials. All of the forth-year medical undergraduates (728) from five representative medical teaching hospitals in Central China were invited to participate in our survey: Tongji Hospital of Tongji Medical College of Huazhong University of Science and Technology, Union Hospital of Tongji Medical College of Huazhong University of Science and Technology, Renmin Hospital of Wuhan University, Zhongnan Hospital of Wuhan University, and Xianning Central Hospital of Hubei University of Science and Technology.

### Survey instrument

The instrument (Additional file 1) was modified from one directed by Abbo et al. (2013). Data collected from the survey included age, sex, education program, clinical rotation completeness, attitude concerning antimicrobial use and resistance, self-perception on preparation for antimicrobial prescribing, rating of the quality of education regarding antimicrobial use, and perception on the antimicrobial knowledge (11 scored questions with different clinical vignettes). Each respondent’s knowledge score was calculated as the sum total of correct answers to each of the 11 knowledge-based questions. The survey asked about which resources (e.g., infectious diseases specialists, hospital pharmacists, and textbooks or study guides) medical students preferred to utilize to determine appropriate anti-infection treatment and involved questions on which study mode they think would be useful to learn about appropriate use of antimicrobials. Responses were single answer among scaled options (mainly 5-level scales plus a ‘not applicable’ option for some items), or free text for some questions.

### Survey administration

Surveys were conducted by directly distributing paper questionnaires to all the fourth-year medical students before their professional classes at the participating sites. The students were instructed to submit their responses a week later in the next class and to abstain from using resources to complete the survey. All the students, with no specific exclusion criteria, responded voluntarily and were anonymous in our study. All of the respondents participated with no incentive. The survey period spanned about 1 month from October 2015 through November 2015.

### Statistical analysis

The answers from the survey forms and the survey sites were de-identified and the survey sites were recorded as “A”, “B”, “C”, “D” and “E”. The completeness of the data were assessed by individual study investigators who were blind to the survey sits. Before the analysis, the 5-level scale responses were merged into dichotomous variables as follows: strongly agree/agree and neutral/disagree/strongly disagree, very useful/useful and neutral/not useful/not at all useful, often/sometimes and rarely/never/not familiar; the 6-level scale responses were dichotomized as the following variables: very good/good and average/poor/very poor/not familiar. As to the knowledge scores of the respondents, the sum of all the correct answers (each counts one point) among the eleven antimicrobial knowledge questions. The data were recorded and analyzed using SPSS 18.0 (SPSS, Inc. Chicago, IL). Nonparametric tests were used to determine and assess the significant difference(s) among the students’ responses from the five survey sites. The Kruskal–Wallis, Mann–Whitney *U* or χ^2^ test were applied to compare the parameters, as appropriate, and the statistical significance was set at P < 0.05. Spearman rank correlation was used to evaluate the effect of student characteristics on their mean knowledge scores. Not all respondents completed all the 11 vignette-based knowledge questions, and adjustment was made to deal with the missing data. The missing values were replaced with the series means before statistical analysis.

## Results

Medical students completed the survey for an overall response rate of 84 % (611/728) (range across teaching hospitals attended, 73–88 %). The mean age was 22 (standard deviation, 0.87), and 43 % (258/606) of the students were male. All of the five teaching hospitals have established antimicrobial stewardship programs. From our results, 170 out of 592 (20 missing) respondents felt they were familiar or very familiar with the term “antimicrobial stewardship” with significant differences among the five teaching hospitals (range across hospitals, 13–51 %, P < 0.001). 12 % (72/588) of the respondents reported having completed a clinical infectious disease rotation during their medical education (range across hospitals, 2–21 %, P < 0.001). Additional file 2 summarizes the characteristics of the respondents and the five participating health care institutions.

### Perceptions and attitudes about antimicrobial use and antimicrobial education

Medical students perceived that antimicrobials are overused and antimicrobial resistance is a problem nationally. Interestingly, fewer students agreed with the existence of these problems in hospitals where they had clinical rotations (Table 1). Overwhelmingly, there was agreement that a strong knowledge of antimicrobials is important for the students’ medical careers (92 %) and inappropriate use of antimicrobials (92 %) can cause antimicrobial resistance and harm patients. It is interesting that fewer thought poor infection-control practices contributes to antimicrobial resistance (71 %) and better use of antimicrobials helps in reducing resistance (86 %). There was significant variability among the medical students’ opinions on some of the issues in Table 1: whether antimicrobials are overused nationally in healthcare (range across hospitals, 78–94 %, P = 0.009), whether a strong knowledge of antimicrobials is important in their medical career (range across hospitals, 86–97 %, P = 0.042), whether inappropriate use of antimicrobials causes antimicrobial resistance (range across hospitals, 84–96 %, P = 0.001) and whether inappropriate use of antimicrobials can harm patients (range across hospitals, 78–95 %, P = 0.03).

**Table 1:** Medical students’ attitudes and perceptions about antimicrobial prescribing and resistance: percentage who agree/strongly agree

Perceptions and attitudes	Agree/strongly agree (%)	P value^b^
	N = 611	
Inappropriate use of antimicrobials causes antimicrobial resistance	92.3	0.03^a^
Strong knowledge of antimicrobials is important in my medical career	91.8	0.042^a^
I would like more education on antimicrobials resistance	90.4	0.339
Better use of antimicrobials will reduce problems with antimicrobial-resistant organisms	86.1	0.225
I would like more education on the appropriate use of antimicrobials	85.9	0.136
Antimicrobials are overused nationally in healthcare	84.8	0.009^a^
Inappropriate use of antimicrobials can harm patients	82.9	0.001^a^
New antimicrobials will be developed in the future that will keep up with the problem of “resistance”	76.9	0.058
Poor infection-control practices by healthcare professionals cause spread of antimicrobial resistance	70.9	0.701
Antimicrobials are overused at the hospitals where I have rotated	39.4	0.151
Prescribing broad-spectrum antimicrobials when equally effective, narrower-spectrum antimicrobials are available increases antimicrobial resistance	32.5	0.579
Appropriate use of antimicrobials can cause antimicrobial resistance	24.6	0.469
Antimicrobial resistance is not a significant problem at the hospitals where I have rotated	15.0	0.101
Antimicrobial resistance is not a significant problem nationally	8.4	0.352

^a^P < 0.05 refers to statistical differences in the percentages of different teaching hospitals

^b^χ^2^ test

The medical students were divided by their preferences for the resources used to acquire the knowledge and information regarding antimicrobial use and resistance (Table 2). The resources included the common approaches to information on antimicrobials, such as textbooks or other study guides (80.2 %), peers (57.7 %), Wikipedia, (52.1 %) and iPhone/smartphone applications (50.5 %). Significant variability among teaching hospitals was found in the students’ use of medical journals, hospital pharmacists, Wikipedia, peers and textbooks or other study guides (P < 0.05). From Table 3, we also found significant differences in the mean knowledge scores between the students who reported using and not using Wikipedia, Johns Hopkins antibiotic guide, IDSA guidelines and infectious diseases specialists. However, regarding to the resources, there were similarities between the knowledge scores of the students who used or did not used them (Table 3).

**Table 2:** Resources used by medical students to learn about antimicrobial use and resistance, percentage reporting resource used sometimes/often

Resource	Sometimes/often (%)	P value^b^
	N = 605	
Textbooks or study guides	80.2	0.012^a^
Peers (other students)	57.7	0.016^a^
Wikipedia	52.1	0.001^a^
iPhone/smartphone applications	50.5	0.489
Non-ID physicians	38.0	0.672
Infectious diseases specialists	27.0	0.322
Hospital pharmacists	22.1	0.031^a^
Medical journals	19.7	<0.001^a^
Other guidelines by professional organizations	17.3	0.726
Pharmaceutical representatives	13.6	0.241
Johns Hopkins Antibiotic Guide	11.5	0.626
Infectious Diseases Society of America guidelines	10.6	0.922
Sanford guide	8.6	0.206

^a^P < 0.05 refers to statistical differences in the percentages of different teaching hospitals

^b^χ^2^ test

**Table 3:** Mean knowledge score for respondents who used the resources compared to respondents who do not use

Resource	Mean knowledge score		P value^b^
	N = 605		
	Used (%)	Not used (%)	
Medical journals	35.3	32.3	0.125
Peers (other students)	35.0	32.8	0.390
Wikipedia	34.8	40.2	<0.001^a^
Textbooks or study guides	34.7	33.9	0.558
iPhone/smartphone applications	34.3	33.7	0.519
Non-ID physicians	33.9	33.0	0.953
Pharmaceutical representatives	33.5	32.9	0.208
Hospital pharmacists	33.4	32.8	0.984
Sanford guide	33.1	33.2	0.664
Other guidelines by professional organizations	33.1	32.7	0.328
Johns Hopkins Antibiotic Guide	32.9	30.3	0.002^a^
Infectious Diseases Society of America guidelines	32.2	29.5	0.04^a^
Infectious diseases specialists	31.0	29.2	0.001^a^

*ID* infectious diseases

^a^P < 0.05 refers to statistical differences in the overall mean scores of respondents who used or not used the resources

^b^Mann–Whitney *U* Test

As to the perceptions of medical education on antimicrobial use and resistance, 408 out of 609 medical students rated their education as useful or very useful, with significant differences between the study sites (range across hospitals, 60–86 %, P < 0.001). Many of the respondents provided replies to an open comment question on the survey emphasized the importance of clinical experience in antimicrobial stewardship. One respondent even suggested, “The courses providing us with knowledge on antimicrobials, should be constantly offered throughout my medical education, besides, they should be better arranged after finishing the curriculum of microbiology and before the study of infectious diseases.” Another student mentioned the importance of infectious disease rotation on learning about appropriate use of antimicrobials, “I had no experience on rotation in infectious disease thus far, so I knew little about the antimicrobials and had not much idea about the answers of the 11 knowledge questions.”The students’ various impressions about their preparedness on appropriate use of antimicrobials are summarized in Table 4.

**Table 4:** Medical students’ perceptions on their education regarding appropriate antimicrobial use and antimicrobial stewardship—percentage who feel their education has been good/very good with comparison across teaching hospitals

Antimicrobial stewardship activity	Good/very good (%)	P value^b^
	N = 606	
Understand the basic mechanisms of antimicrobial resistance	54.1	<0.001^a^
Know when to start antimicrobial therapy	40.0	0.004^a^
Select an appropriate regimen	36.9	0.021^a^
Describe the correct spectrum of antimicrobial therapy for different antimicrobials (what is covered by each drug)	35.2	0.009^a^
Transition from intravenous to oral antimicrobials (intravenous to oral switch)	34.2	<0.001^a^
Find reliable sources of information to treat infections	34.0	<0.001^a^
Interpret antibiograms	28.3	<0.001^a^
Streamline or deescalate antimicrobial therapy	28.2	0.040^a^
Handle a patient who demands antimicrobial therapy that is not indicated	25.6	0.254

^a^P < 0.05 refers to statistical differences in the percentages of different teaching hospitals

^b^χ^2^ test

### Education resources and knowledge

Of the 608 respondents, 152 (25 %) recalled having courses about appropriate use of antimicrobials, 97 (16 %) about when to start using antimicrobials, 107 (18 %) about how to select the proper duration of antimicrobial treatment for specific infections, and 88 (14 %) about how to select the correct doses of antimicrobials. Table 5 shows the results from the knowledge assessment section of the survey. The general mean knowledge score of the medical students across the 11 items was 3.78 (total percentage correct, 34 %), with a standard deviation of 1.57 and statistically significant differences across medical colleges (range across hospitals, 31–40 %, P < 0.001). Our analyses showed that the knowledge score had a significant, yet weakly positive correlation with having had an infectious diseases clinical elective rotation (Spearman correlation coefficient R = 0.095, P < 0.05) and rating their medical education as useful or very useful (Spearman correlation coefficient R = 0.088, P < 0.05).

**Table 5:** Summary of knowledge vignettes with the corresponding percentage of correct answers with comparison across teaching hospitals

Clinical vignette	Percentage correct (%)	P value^b^
	N = 595	
Complicated UTI: appropriate antimicrobial selection and duration of treatment	52.3	<0.001^a^
Recognize the spectrum of activity of selected antimicrobial agents	22.3	0.114
Diagnosis of community acquired pneumonia: selection of appropriate antimicrobial and switch intravenous to oral therapy	45.0	0.017^a^
Recognize Clostridium difficile infection secondary to the use of antimicrobials	89.4	0.025^a^
Recognize the possible risks associated with unnecessary use of antimicrobials	50.7	<0.001^a^
Match the antimicrobial/organism with most likely mechanism of resistance		
*E. coli*/β-lactam resistance	39.1	<0.001^a^
*S. aureus*/methicillin resistance	37.0	0.326
*S. aureus*/vancomycin intermediate	27.5	0.032^a^
Enterococcus/cephalosporin	11.9	0.018^a^
Extended spectrum β-lactamase positive *E. coli* bacteremia: antimicrobial selection	5.0	0.1
Identify scenarios with potential for unnecessary use of antimicrobials	5.0	0.439

*UTI* urinary tract infection

^a^P < 0.05 refers to statistical differences in the percentages of different teaching hospitals

^b^Kruskal–Wallis test

## Discussion

In our study, we assessed the attitudes and perceptions of fourth year medical students in regard to appropriate antimicrobial prescribing and resistance across five representative teaching hospitals in Central China. The vast majority of students in this study believed that better use of antimicrobials will reduce problems with antimicrobial-resistant organisms, and that more education on appropriate use of antimicrobials was desired. Many students showed their concerns with the problem that antimicrobials were overused in healthcare nationally, but fewer students were aware of that problem in the institutions where they had clinical rotations, which was similar to the results observed in other reports (Abbo et al. 2013; Minen et al. 2010). The respondents were more divided on whether inappropriate use of antimicrobials can harm patients (P = 0.001).

A variety of resources were reported by the medical students to learn about antimicrobial use and resistance. The resources frequently used by the Chinese medical students were textbooks/study guides (80 %), peers (58 %), Wikipedia (52 %) and smartphone applications (50 %). There was no significant difference on mean scores between the students who used and not used textbooks, peers or smartphone applications. It seems that these frequently used resources did not make difference to the knowledge scores of the students who used them. Obviously, Wikipedia, peers and smartphone applications were easily available resources, but not professional ones to the medical students. The textbooks or study guides, covering more basic knowledge but less cutting-edge information, could not enable the students to progress to further learning of the knowledge regarding antimicrobials use and resistance. Interestingly, the infrequently used resources, Johns Hopkins antibiotic guide (11 %), IDSA guidelines (11 %) and infectious diseases specialists (27 %), were significantly related to the enhancement of the mean scores (P < 0.05). Although these US published materials were not easily available for most Chinese students (expensive or hard to read) and were not listed as required references to the students by the attended institutions, the students who used them were able to acquire the cutting-edge knowledge regarding current clinical antimicrobial therapy. Given the limited usage of the US published materials, consideration should be given to improving accessibility of foreign professional published materials in Chinese libraries.

In our study, not many students (generally less than half) believed their education on specific stewardship activities was very good or good. Significant difference was observed across hospitals in perceived educational value. In regard to the knowledge assessment section, students got less than half of the questions correct (34 %) with significant variability among teaching hospitals. The majority (88 %) of the respondents had not completed a clinical infectious rotation when they gave feedback to our survey, and felt that their medical institutions should offer more educational activities on the topic of antimicrobial stewardship by means of both classroom lectures and clinical rotations. These data highlight the opportunity for increased education in infectious diseases, especially considering the limited clinical rotation training.

Because our survey was designed similarly to some studies of medical students from the United States, some interesting comparisons (although these comparisons do not use inferential statistics) can be made (Abbo et al. 2013; Minen et al. 2010). Primarily, our medical college students’ attitudes toward antimicrobial use and resistance bore a resemblance with those of the US students. Besides, regarding to the references for antimicrobial use and resistance study, Chinese medical students were more likely to rely on textbooks/study guides (80 vs. 46 % reported as often or sometimes used) and Wikipedia (52 vs. 41 %)while the US students’ choices tended to be handheld devices/smartphone apps, hospital pharmacists and physicians. Peers (58 vs. 54 %) were the frequently used resources in our study and Abbo et al’s study (not included as an option in Minen et al’s study), while pocket guidelines (e.g. IDSA guidelines) and pharmaceutical representatives were the infrequently used ones in all the studies. In addition, both the US and Chinese students had a comfortable understanding of the basic mechanisms of antimicrobial resistance, but the Chinese students felt less comfortable with finding reliable sources of information to treat infections and handling patients demanding unnecessary antibiotics than the US students. Thus, it may be that the optimal antimicrobial stewardship program should include more clinic practical training and less classroom learning. The Chinese students acquired poorer knowledge scores than the US students (34 vs. 51 %), despite their favorably ratings of the usefulness of their antimicrobial education (67 vs. 58 % reported as useful or very useful). The knowledge vignettes with a wide variance of correct responses were antimicrobial selection to extended spectrum β-lactamase positive *E. coli* bacteremia (5 % of the Chinese students answered correctly vs. 32 % of the US students), identification of the scenarios with the potential for unnecessary use of antimicrobials (5 % vs. 59 %), diagnosis of community acquired pneumonia (45 vs. 87 %), and recognition of the possible risks associated with the unnecessary use of antimicrobials (15 vs. 91 %). Nevertheless, the results may be biased by the fact that the fourth-year medical education might be different between China and the USA, in terms of lectures and training. These data highlight the gap between Chinese students’ perceptions regarding antimicrobials and infectious diseases and those of the US students. Efforts from Chinese medical education institutions should be made to increase students’ awareness of proper antimicrobial prescribing practices.

The major strength of our study was that this survey was anonymous and voluntary, which might encourage the respondents give authentic answers rather than socially desirable ones. We had high response rates (84 %), and the participants hailed from well-established medical institutions. This study has several inherent limitations. Less detailed data were collected regarding population composition among the student characteristics, such as anticipated postgraduate-training directions and interests in ID specialty. In addition, all of the data were voluntarily reported, and no information was obtained regarding the students who did not reply to the survey. Our study only involved medical students across 5 teaching hospitals in Central China., so it was quite possible that the responsive and non-responsive students may share common demographic characteristics, alleviating selection bias in some ways (Halbesleben and Whitman 2013; Johnson and Wislar 2012). Finally, the clinical vignettes covered only a sample of the important knowledge regarding antimicrobials, and the students might have used external resources to answer their questionnaires, thus providing a better estimate of their knowledge scores.

## Conclusion

This study shows the medical students’ desires for further education on antimicrobial use and resistance, and antimicrobial stewardship programs was generally rated favorably by the students. However, the medical students’ attitudes to the usefulness of their medical education did not match their mean knowledge scores, which were assessed through a series of clinical vignettes. These results reveal an obvious gap between the medical students’ perceptions on antimicrobial prescribing from China and the United States. Solutions to address this gap could be continuous medical education on antimicrobial prescribing and reformation of medical curriculum setting. Further investigations and studies are necessary to focus on methods for enhancing medical students’ perceptions on antimicrobial use and resistance and improving prudent antimicrobial prescribing in hospitals.

## Additional files

10.1186/s40064-016-3454-0 Questionnaire. The questionnaire in our study included items concerning age, sex, education program, clinical rotation completeness, attitudes concerning antimicrobial use, understanding of antimicrobial resistance and so on.

10.1186/s40064-016-3454-0 Baseline Characteristics of Hospitals and Respondents by Participating Teaching Hospitals. Characteristics of the teaching hospitals and the respondents attended in our study.

## Abbreviations

CDC: Centers for Disease Control
GNB: gram-negative bacteria
MRSA: methicillin-resistant *Staphylococcus aureus*
VRSA: vancomycin-resistant *Staphylococcus aureus*
WHO: World Health Organization

## References

[CR1] Abbo LM, Cosgrove SE, Pottinger PS, Pereyra M, Sinkowitz-Cochran R, Srinivasan A (2013). Medical students’ perceptions and knowledge about antimicrobial stewardship: how are we educating our future prescribers?. Clin Infect Dis.

[CR2] Afzal Khan AK, Banu G, Reshma KK (2013). Antibiotic resistance and usage-a survey on the knowledge, attitude, perceptions and practices among the medical students of a Southern Indian Teaching Hospital. J Clin Diagn Res.

[CR3] Centers for Disease Control and Prevention (CDC) (2013) Antibiotic resistance threats in the United States. http://www.cdc.gov/drugresistance/pdf/ar-threats-2013-508.pdf. Accessed 16 Sep 2016

[CR4] Centers for Disease Control and Prevention (CDC) (2014) Core elements of hospital antibiotic stewardship programs. http://www.cdc.gov/getsmart/healthcare/pdfs/checklist.pdf. Accessed 16 Sep 2016 10.1093/cid/ciu542 PMC6521960 25261548

[CR5] Charani E, Cooke J, Holmes A (2010). Antibiotic stewardship programmes–what’s missing?. J Antimicrob Chemother.

[CR6] Dyar OJ, Howard P, Nathwani D, Pulcini C (2013). Knowledge, attitudes, and beliefs of French medical students about antibiotic prescribing and resistance. Med Mal Infect.

[CR7] Halbesleben JR, Whitman MV (2013). Evaluating survey quality in health services research: a decision framework for assessing nonresponse bias. Health Serv Res.

[CR8] Hecker MT, Aron DC, Patel NP, Lehmann MK, Donskey CJ (2003). Unnecessary use of antimicrobials in hospitalized patients: current patterns of misuse with an emphasis on the antianaerobic spectrum of activity. Arch Intern Med.

[CR9] Huang Y, Gu J, Zhang M, Ren Z, Yang W, Chen Y (2013). Knowledge, attitude and practice of antibiotics: a questionnaire study among 2500 Chinese students. BMC Med Educ.

[CR10] Ibia E, Sheridan M, Schwartz R (2005). Knowledge of the principles of judicious antibiotic use for upper respiratory infections: a survey of senior medical students. South Med J.

[CR11] Johnson TP, Wislar JS (2012). Response rates and nonresponse errors in surveys. JAMA.

[CR12] Li L, Duan Y, Chen P, Li J, Mao X, Barraclough BH (2012). Knowledge, skills, and attitudes of medical students to patient safety: a cross-sectional pilot investigation in China. J Evid Based Med.

[CR13] Luther VP, Ohl CA, Hicks LA (2013). Antimicrobial stewardship education for medical students. Clin Infect Dis.

[CR14] Lv B, Zhou Z, Xu G, Yang D, Wu L, Shen Q (2014). Knowledge, attitudes and practices concerning self-medication with antibiotics among university students in western China. Trop Med Int Health.

[CR15] Minen MT, Duquaine D, Marx MA, Weiss D (2010). A survey of knowledge, attitudes, and beliefs of medical students concerning antimicrobial use and resistance. Microb Drug Resist.

[CR16] Owens RC, Fraser GL, Stogsdill P (2004). Antimicrobial stewardship programs as a means to optimize antimicrobial use. Insights from the Society of Infectious Diseases Pharmacists. Pharmacotherapy.

[CR17] Paterson DL (2006). The role of antimicrobial management programs in optimizing antibiotic prescribing within hospitals. Clin Infect Dis.

[CR18] Pulcini C, Wencker F, Frimodt-Møller N, Kern WV, Nathwani D, Rodríguez-Baño J (2015). European survey on principles of prudent antibiotic prescribing teaching in undergraduate students. Clin Microbiol Infect.

[CR19] Review on Antimicroibal Resistance (2016) Tackling drug-resistant infections globally: final report and recommendations. http://amr-review.org/sites/default/files/160525_Final%20paper_with%20cover.pdf. Accessed 16 Sep 2016

[CR20] Thriemer K, Katuala Y, Batoko B, Alworonga JP, Devlieger H, Van Geet C (2013). Antibiotic prescribing in DR Congo: a knowledge, attitude and practice survey among medical doctors and students. PLoS One.

[CR21] Wilcox MH (2009). The tide of antimicrobial resistance and selection. Int J Antimicrob Agents.

[CR22] World Health Organization (WHO) (2012) The evolving threat of antimicrobial resistance—options for action. http://apps.who.int/iris/bitstream/10665/44812/1/9789241503181_eng.pdf. Accessed 16 Sep 2016

