# Supplementary material for: Attitudes and perceptions regarding antimicrobial use and resistance among medical students in Central China
Source: Springerplus. 2016 Oct 12;5(1):1779. doi: 10.1186/s40064-016-3454-0 (PMC5061672; doi:10.1186/s40064-016-3454-0)
Supplement: Supplementary file 1 — 10.1186/s40064-016-3454-0 Questionnaire. The questionnaire in our study included items concerning age, sex, education program, clinical rotation completeness, attitudes concerning antimicrobial use, understanding of antimicrobial resistance and so on. [file 40064_2016_3454_MOESM1_ESM.doc]

Additional file S1

This survey will assess 4th year medical students' knowledge and attitudes about antimicrobial use and resistance as well as the quantity and perceived quality of education about antimicrobials in a sample of Chinese teaching hospitals. Our ultimately aim was to identify areas of potential improvement in medical college curricula.

Your participation in this survey is completely voluntary. Your responses will be anonymous (de-identified) and will only be seen individually by the study investigators. Responses will be aggregated for general release. This survey will not collect any personally identifiable data other than age and gender. We will try to make sure that the information we collect from you is kept private and used only for this research study. Completing this questionnaire will serve as your consent to take part in this research study.

The survey should take about 20 minutes to complete. Please take the survey only once. You should better complete all the questions in this questionnaire, but you may stop this study at any time without any penalty to you. We understand it is tempting to "look up answers" but PLEASE do not use references and answer honestly the best you can.

Whether or not taking part in this survey will make no difference to your academic grade or rating. Completing this questionnaire will serve as your consent to take part in this research study.

Thank you for your time and participation!

| Please tell us about yourself: | | | | | | | | | | | |
| --- | --- | --- | --- | --- | --- | --- | --- | --- | --- | --- | --- |
| 1. Tell us your age | | | | | | | | | | | |
|  |  |  | |  |  | |  | |  | | |
|  |  |  | |  |  | |  | |  | | |
| 2. Please tell us your gender | | | | | | | | | | | |
| ○ Female | ○ Male |  | |  |  | |  | |  | | |
|  |  |  | |  |  | |  | |  | | |
| 3. Tell us how much you Agree or Disagree with each of the following statements | | | | | | | | | | | |
|  | | | Strong Agree | | | Agree | | Neutral | | Disagree | Strong Disagree |
| Antimicrobials are overused nationally | | |  | | |  | |  | |  |  |
| Antimicrobials are overused at the hospitals where I have rotated | | |  | | |  | |  | |  |  |
| Antimicrobial resistance is not a significant problem nationally | | |  | | |  | |  | |  |  |
| Antimicrobial resistance is not a significant problem at the hospitals where I have rotated | | |  | | |  | |  | |  |  |
| Better use of antimicrobials will reduce problems with antimicrobial resistant organisms | | |  | | |  | |  | |  |  |
| Appropriate use of antimicrobials can cause antimicrobial resistance | | |  | | |  | |  | |  |  |
| Strong knowledge of antimicrobials is important in my medical career | | |  | | |  | |  | |  |  |
| I would like more education on antimicrobial resistance | | |  | | |  | |  | |  |  |
| I would like more education on the appropriate use of antimicrobials | | |  | | |  | |  | |  |  |
| New antimicrobials will be developed in the future that will keep up with the problem of “resistance” | | |  | | |  | |  | |  |  |
| Prescribing broad spectrum antimicrobials when equally effective narrower spectrum antimicrobials are available increases antimicrobial resistance | | |  | | |  | |  | |  |  |
| Inappropriate use of antimicrobials causes antimicrobial resistance | | |  | | |  | |  | |  |  |
| Inappropriate use of antimicrobials can harm patients | | |  | | |  | |  | |  |  |

| 4. If made available to you, please rate the usefulness of each of the following options for learning about antimicrobial prescribing and resistance? | | | | | | |
| --- | --- | --- | --- | --- | --- | --- |
|  | Very Useful | Useful | Neutral | Not Useful | Not at all Useful | N/A |
| Grand Rounds Lectures |  |  |  |  |  |  |
| Lecture series for medical students |  |  |  |  |  |  |
| Interactive patient oriented problem solving modules on the internet |  |  |  |  |  |  |
| Interactive patient oriented problem solving modules on CD-ROM |  |  |  |  |  |  |
| Problem solving sessions attended by small groups of medical students and residents or faculty |  |  |  |  |  |  |
| Roleplaying sessions dealing with patients demanding sessions dealing with patients demanding |  |  |  |  |  |  |

| 5. How do you use each of the following sources to learn about antimicrobial use and resistance? Please rate each of the following options | | | | | |
| --- | --- | --- | --- | --- | --- |
|  | Often | Sometimes | Rarely | Never | Not Familiar |
| Infectious Diseases specialists |  |  |  |  |  |
| Non infectious diseases physicians |  |  |  |  |  |
| Infectious Diseases Society of America Guidelines |  |  |  |  |  |
| Other Guidelines by professional organizations |  |  |  |  |  |
| Hospital Pharmacists |  |  |  |  |  |
| Pharmaceutical representatives |  |  |  |  |  |
| Medical journals |  |  |  |  |  |
| Sanford guide |  |  |  |  |  |
| Johns Hopkins Antibiotic guide |  |  |  |  |  |
| iPhone or smartphone apps |  |  |  |  |  |
| UptoDate |  |  |  |  |  |
| Wikipedia |  |  |  |  |  |
| Textbooks or study guides |  |  |  |  |  |
| Peers (other students) |  |  |  |  |  |
| Others used often (please specify) |  | | | | |

6. Have you completed a clinical rotation in Infectious Diseases during medical school?

○ Yes

○ No

| 7. Have you attended any formal lecture(s) that address the following topics during medical school? | | | |
| --- | --- | --- | --- |
|  | Yes | No | I don't remember |
| Rational use of antibiotics in general |  |  |  |
| When to start antibiotics |  |  |  |
| How to select the correct dosing |  |  |  |
| How to select the right duration of treatment for specific infections |  |  |  |

8. How would you rate your education regarding appropriate use of antimicrobials so far?

○ Very useful

○ Useful

○ Neutral

○ Not Useful

○ Not at all useful

○ I have not received any education so far"

| 9. How well do you feel that your medical education has prepared you to do the following upon graduation? | | | | | | |
| --- | --- | --- | --- | --- | --- | --- |
|  | Very Good | Good | Average | Poor | Very Poor | Not Familiar |
| To know when to start antimicrobial therapy |  |  |  |  |  |  |
| How to select the best antimicrobial for a specific infection |  |  |  |  |  |  |
| To describe the correct spectrum of antimicrobial therapy for different antimicrobials(what is covered by each drug) |  |  |  |  |  |  |
| Understand the basic mechanisms of antimicrobial resistance |  |  |  |  |  |  |
| How to streamline or deescalate antimicrobial therapy |  |  |  |  |  |  |
| How to interpret antibiograms |  |  |  |  |  |  |
| How to find reliable sources of information to treat infections |  |  |  |  |  |  |
| How to transition from intravenous to oral antibiotics (IV to PO switch) |  |  |  |  |  |  |
| How to handle a patient who demands antimicrobial therapy that is not indicated |  |  |  |  |  |  |

10. When do you think your medical school should spend more time teaching about the appropriate use of antimicrobials?

○ First year

○ Second year

○ Third year

○ Fourth year

○ All of the above

○ None of the above

11. A 50 year old woman presents to clinic with 3 days of dysuria and feeling feverish. On exam she has a temperature of 101.8 F, her blood pressure and heart rate are normal and at her baseline. There is mild costovertebral angle tenderness and suprapubic tenderness. She is menopausal but sexually active with only one male partner. Labs are ordered, including a urinalysis showing 80 white cells/high-powered field and large leukocyte esterase and nitrates; culture is pending. Renal function is normal. She has never taken antibiotics before and there is no documented antimicrobial resistance to urinary pathogens in this area.

You would:

○ Treat with nitrofurantoin for 14 days

○ Treat with trimethoprim/ sulfamethoxazole (Bactrim) for 14 days

○ Treat with ciprofloxacin, adjusting if the organism is resistant, and plan for a 7 day treatment course

○ Treat with vancomycin and piperacillin/tazobactam (Zosyn)

○ Treat with vancomycin alone

○ Await culture results before starting an antimicrobial

○ Refer to a urologist

12. Which of the following antimicrobials do not cover anaerobes?

○ Metronidazole (Flagyl)

○ Clindamycin (Cleocin)

○ Ampicillin/Sulbactam (Unasyn)

○ Piperacillin/Tazobactam (Zosyn)

○ Levofloxacin (Levaquin)

13. A 28 year old man is hospitalized with fevers, chills and productive cough, a CXR reveals a RLL infiltrate. You make the diagnosis of community acquired pneumonia. The resident on call starts IV levofloxacin. After 24 hours he feels better and his fever is improving, but he still has a productive cough. Blood and sputum cultures reveal Streptococcus pneumoniae (resistant to penicillin) and susceptible to fluoroquinolones. He is able to eat, oral absorption is good. With regards to antimicrobial therapy, what would you do next?

○ Continue intravenous levofloxacin (Levaquin)

○ Switch to oral levofloxacin (Levaquin)

○ Switch to ampicillin/ sulbactam (Unasyn)

○ Switch to piperacillin/ tazobactam (Zosyn)

○ Add vancomycin

○ Switch to vancomycin plus piperacillin/tazobactam (Zosyn)

| 14. A 14 year old girl comes to the clinic complaining of a sore throat that started the previous day. Two of her friends have the same symptoms. She has no fever, chills, cough, runny nose, itchy eyes. She has no allergies and currently is not taking any medications.   On exam she is not acutely ill; T 37.2 C; HR 70/min; respiratory rate 12/min; BP 110/70 mmHg; no cough; small amount of exudate on tonsils; small cervical nodes; few scattered petechiae on her palate; no rash.   Based on her presenting history, signs and symptoms, please select how likely are each of the following options: | | | | | |
| --- | --- | --- | --- | --- | --- |
|  | Most Likely | Likely | Neutral | Unlikely | Very Unlikely |
| Bacterial pharyngitis |  |  |  |  |  |
| Viral upper respiratory infection |  |  |  |  |  |
| Fungal pharyngitis |  |  |  |  |  |
| Asthma exacerbation |  |  |  |  |  |
| Pneumonia |  |  |  |  |  |
| Seasonal allergy |  |  |  |  |  |

15. Which of the following would you do next? Please select one

○ Throat swab for culture and sensitivity

○ Throat swab for culture and give her a prescription for antibiotics while waiting for the results

○ Blood sample for culture and sensitivity

○ Prescription for antibiotic

○ Start her on antibiotics immediately with a supply from your office

○ Chest x-ray

○ Admit to the hospital

16. The patient gets a prescription for 10 days of ciprofloxacin from a neighbor. Within 5 days of taking the antibiotic she presents to the ER with fever and more than 8 watery bowel movements over 12 hours. What do you think is the most likely cause of her diarrhea?

○ Food poisoning

○ Antibiotic associated diarrhea

○ Clostridium difficile

○ Typhoid fever

○ Non typhoid salmonellosis associated with food contamination

1. A 78 year old gentleman is hospitalized for elective knee replacement surgery. The orthopedist requests 5 days of postoperative prophylactic antibiotics. What are the possible risks associated with prescribing 5 days of antibiotics in this case?

○ Antibiotic resistance

○ It reinforces the perception that long courses of antibiotics should be prescribed for surgical prophylaxis

○ Adverse drug reaction

○ Clostridium difficile associated diarrhea

○ All of the above

○ No real risk

1. For each of the following combinations of antimicrobials/organisms please match the most likely mechanism of antimicrobial resistance.

|  | Efflux pumps | Alteration of binding site | Thickening of the cell wall | Enzymatic | Intrinsic (not acquired) |
| --- | --- | --- | --- | --- | --- |
| Beta-lactam resistance in E.coli |  |  |  |  |  |
| Methicillin resistant Staphylococcus aureus (MRSA) |  |  |  |  |  |
| Vancomycin intermediate Staphylococcus aureus (VISA) |  |  |  |  |  |
| Cefazolin resistance in enterococcus |  |  |  |  |  |

19. The lab calls you with a positive blood culture for extended spectrum beta lactamase positive (ESBL) E.coli. Which one is your preferred empiric antibiotic to treat this bacteremia?

○ Daptomycin (Cubicin)

○ Vancomycin

○ Piperacillin/tazobactam (Zosyn)

○ Vancomycin and piperacillin/tazobactam (Zosyn)

○ Ceftriaxone (Rocephin)

○ Micafungin (Mycamine)

○ Meropenem (Merrem)

1. According to Centers for Disease Control and Prevention (CDC) guidelines, intravenous vancomycin use is discouraged in which of the following situations?

○ For the eradication of MRSA colonization

○ For the treatment of Methicillin Susceptible Staphylococcus aureus (MSSA) bacteremia in a patient on hemodialysis

○ Treatment of 1 out of 4 positive blood cultures with Staphylococcus haemolyticus in a patient with no central lines

○ All of the above

○ None of the above

21. Are you familiar with the term "Antimicrobial Stewardship"?

○ Very familiar

○ Familiar

○ Not familiar (I've heard the term but not sure what it is about))

○ Not at all familiar (I've never heard it before)

○ No

○ I've heard about it but not sure what it is about
