# Supplementary material for: Attitudes and perceptions regarding antimicrobial use and resistance among medical students in Central China
Source: Springerplus. 2016 Oct 12;5(1):1779. doi: 10.1186/s40064-016-3454-0 (PMC5061672; doi:10.1186/s40064-016-3454-0)
Supplement: Supplementary file 2 — 10.1186/s40064-016-3454-0 Baseline Characteristics of Hospitals and Respondents by Participating Teaching Hospitals. Characteristics of the teaching hospitals and the respondents attended in our study. [file 40064_2016_3454_MOESM2_ESM.doc]

**Additional file S2**

**Baseline Characteristics of Hospitals and Respondents by Participating Teaching Hospitals**

| **Characteristics of Teaching Hospitals** | **Participating Teaching Hospitals** | | | | |  |
| --- | --- | --- | --- | --- | --- | --- |
|  | **A** | **B** | **C** | **D** | **E** |  |
| Response Rate, % (n/N) | 87.12(115/132) | 73.33(77/105) | 81.39(70/86) | 77.78(63/81) | 88.27(286/324) |  |
| Type of course(s) teaching AS | Required | Required | Elective | Elective | Required |  |
| **Characteristics of Respondents** |  |  |  |  |  | **P value** |
| Age, (Mean ±SD) | 21.72± 0.87 | 21.52 ± 0.99 | 21.67 ± 0.94 | 21.24 ± 1.03 | 21.88 ± 0.97 | <0.001 |
| Completed ID rotation | 2/115(1.74%) | 1/77(1.30%) | 9/67(13.43%) | 3/61(4.92%) | 57/268(21.27%) | <0.001 |
| AS: Antimicrobial Stewardship; ID, infectious diseases. | | | | | | |
